# The complex contribution of NOS interneurons in the physiology of cerebrovascular regulation

**DOI:** 10.3389/fncir.2012.00051

**Published:** 2012-08-09

**Authors:** Sonia Duchemin, Michaël Boily, Nataliya Sadekova, Hélène Girouard

**Affiliations:** Department of Pharmacology, Université de MontréalMontreal, QC, Canada

**Keywords:** astrocyte, autoregulation, cerebral blood flow, GABA, interneuron, magnetic resonance imaging, neurovascular coupling, nitric oxide

## Abstract

Following the discovery of the vasorelaxant properties of nitric oxide (NO) by Furchgott and Ignarro, the finding by Bredt and coll. of a constitutively expressed NO synthase in neurons (nNOS) led to the presumption that neuronal NO may control cerebrovascular functions. Consequently, numerous studies have sought to determine whether neuraly-derived NO is involved in the regulation of cerebral blood flow (CBF). Anatomically, axons, dendrites, or somata of NO neurons have been found to contact the basement membrane of blood vessels or perivascular astrocytes in all segments of the cortical microcirculation. Functionally, various experimental approaches support a role of neuronal NO in the maintenance of resting CBF as well as in the vascular response to neuronal activity. Since decades, it has been assumed that neuronal NO simply diffuses to the local blood vessels and produce vasodilation through a cGMP-PKG dependent mechanism. However, NO is not the sole mediator of vasodilation in the cerebral microcirculation and is known to interact with a myriad of signaling pathways also involved in vascular control. In addition, cerebrovascular regulation is the result of a complex orchestration between all components of the neurovascular unit (i.e., neuronal, glial, and vascular cells) also known to produce NO. In this review article, the role of NO interneuron in the regulation of cortical microcirculation will be discussed in the context of the neurovascular unit.

## Introduction

Nitric oxide (NO) is a small inorganic, labile gaseous molecule originally identified as endothelium-derived relaxing factor (EDRF) mediating relaxation of blood vessels (Furchgott and Zawadzki, 1980). NO is produced through the enzymatic conversion of L-arginine to L-citrulline by the enzyme NO synthase (NOS). NOS includes three main isoforms: the constitutive endothelial eNOS and neuronal nNOS as well as the inducible iNOS. The discovery by Bredt and colleagues (Bredt and Snyder, 1989) that NO is also produced in the brain led to the finding that NO could act as a neurotransmitter and a modulator of cerebral blood flow (CBF).

CBF is regulated by two main mechanisms: autoregulation and neurovascular coupling (NVC). CBF autoregulation is the primary mechanism ensuring that the flow and supply of oxygen, glucose, and nutrients through the vascular beds remain within the upper and lower limits of the autoregulatory range (50–160 mm Hg) during fluctuations in systemic arterial pressure. NVC is the dynamic link between neuronal energy needs and hemodynamic changes. In the cortex, NVC depends on the complex interplay between neurons, astrocytes, and microvessels (endothelial cells, myocytes, and pericytes) that form the “neurovascular unit” (Iadecola, 2004). NO is known to play a pivotal role in mechanisms underlying both autoregulation and NVC. However, although many studies have attempted to elucidate the role of NO in the CBF regulation, the exact source of NO involved at different levels of CBF regulation as well as its molecular and cellular targets remains unresolved. This review article will focus on the physiological role of a specific subsets NO producing gamma aminobutyric acid (GABA)ergic neurons, the NOS interneurons in control of cortical CBF. The current knowledge on this topic will be critically examined in the context of the neurovascular unit.

## Anatomical relationship between no interneurons and blood vessels

The intracortical NOS interneurons are part of a family of GABA inhibitory interneurons believed to play a pivotal role in CBF regulation. Indeed, NOS interneurons have been demonstrated to be ideally positioned between glutamatergic pyramidal cells and local microvessels (Cauli et al., 2004). Most anatomical studies on NOS interneurons have used immunohistochemistry for the nicotinamide adenine dinucleotide phosphate-diaphorase (NADPHd) which constitutes a histochemical marker for nNOS. This method is prefered for use due to its simplicity compared to NOS immunohistochemistry and *in situ* hybridization since it requires only two reagents, nitro blue tetrazolium and NADPH. Histochemistry for NADPHd is believed to be very specific for nNOS in aldehyde-fixed mammalian brain tissue (Matsumoto et al., 1993). Firstly, in the neocortex of many species the overlap of immunocytochemical staining of nNOS and NADPHd is quasi absolute in neurons. Secondly, *in situ* hybridization for nNOS mRNA in combination with NADPHd show that each positive cell for NADPHd in rat cerebral cortex also exhibits autoradiographic staining for nNOS mRNA (Bredt et al., 1991). Thirdly, the deletion of the gene coding for nNOS results in the absence of NADPHd staining in mice nervous sytem (Huang et al., 1993).

Cortical NADPHd neurons are divided in type I and type II neurons according to the intensity of staining. Type I cells exhibit large somata and are intensely stained while type II cells are much smaller and weakly stained (Kubota et al., 1994; Yan et al., 1996). Type I neurons are found in the cortex of various species including mouse, cat, monkey, and humans. Their distribution pattern is similar between species and are found in all cortical layers (Sandell, 1986; Mizukawa et al., 1988; Oermann et al., 1999; Garbossa et al., 2005). The numerical density of type I cells is lower in the monkey than in the rat (Yan et al., 1994). Type II cells have a smaller soma and lower NADPHd activity, and are 20-fold more numerous than type I cells in primates. Type II cells are found mainly in the supragranular layers in monkey (Yan et al., 1996) and human (Judas et al., 1999) while, in rodents, they are about twofold more numerous than type I cells and populate all neocortical layers (Perrenoud et al., 2012b). In primates, pyramidal cells also present some NADPHd reactivity or nNOS immunostaining in different cortical areas (Barone and Kennedy, 2000; Garbossa et al., 2005). Although the association of type I interneurons with blood vessels have been recently described in the adult monkey (Rockland and Nayyar, 2012) the association of type II neurons with blood vessels remain to be described.

Around 80% of NADPHd positive cells in the rat cortex contain GABA and they account for 2% of the GABAergic cells (Valtschanoff et al., 1993) which represent about 15% of cortical neurons in rodents (Gabbott et al., 1997). NADPHd-positive interneurons co-express many vasoactive mediators such as GABA, neuropeptide Y (NPY), somatostatin (SOM), and calbindin (Kummer et al., 1992; Kubota et al., 1994; Xiao et al., 1996; Abounader and Hamel, 1997; Gonchar and Burkhalter, 1997; Estrada and DeFelipe, 1998). Indeed, using patch-clamp recordings, biocytin labeling, and single-cell reverse transcriptase-PCR, Karagiannis et al. (2009), showed that nNOS was expressed by 9% of fast spiking parvalbumin (PV)-interneurons, 6% of adapting SOM-interneurons, 2% of adapting vasoactive intestinal peptide (VIP)-interneurons, 0% of bursting VIP-interneurons, and 26% of adapting NPY-interneurons. More recently, double labeling studies showed colocalisation of cytochrome P450 2C11 epoxygenase and soluble epoxygenase with nNOS within perivascular nerves which suggests synthesis of the vasodilator eicosatrienoic acids in nitrergic nerves (Iliff et al., 2007). The authors concluded that both the P450 epoxygenase and NOS pathways seem to be involved in the local CBF response to N-methyl-D-aspartate (NMDA) receptor activation. As in every neuron, NOS interneurons release potassium (K^+^), hydrogen ions, and adenosine produced by ATP catabolism in response to neuronal activity (Iliff et al., 2003).

In addition to releasing various vasoactive mediators, nitrergic nerves are strategically positioned in proximity to cerebral arteries. NADPHd positive fibers have been found around pial arteries as well as parenchymal vessels. Pial arteries are innervated by perivascular nitrergic nerves that originate from sphenopalatine, otic, and trigeminal ganglia (Suzuki et al., 1994), while fibers close to parenchymal arteries have been identified as GABAergic interneurons. NOS interneurons have access to parenchymal arterioles but apparently not to arterioles and arteries proximal to the Virchow-Robin space (Abadia-Fenoll, 1969; Busija, 1993). Using electron microscopy, it has been demonstrated that the axons, dendrites, or somata of NADPHd positive neurons contact the basal membrane of blood vessels or the perivascular astroglia (Vaucher et al., 2000). As a matter of fact, a very specific neurovascular connection has been observed in isoled cortical parenchymal arteries where a network of fibers or individual fibers is attached along isolated vessels (Estrada et al., 1988). In a very elegant study, Cauli et al. (2004) identified the subtypes of interneurons associated with rat cortical microvessels in layer I–III. According to this study, at a distance within 50 μm of the blood vessels, the percentage of GABAergic neuron subsets appears to be the following: 39% express VIP or NPY, 28% express NOS, 28% express SOM, some cells co-express more than one marker. This distribution differed considerably with their respective density in the same layers and fields of the somatosensory cortex, which was VIP (46.1%) > SOM (30.4%) > NPY (16.1%) > NOS (7.4%). These results indicate a privileged redistribution of NPY and NOS interneurons in the vicinity of cortical microvessels. Indeed several authors have reported that cortical nitrergic neurons are localized in apposition to cerebral arteries, especially in bifurcation areas surrounding vessels with their projections or sending projections to more distant arterioles and capillaries (Estrada et al., 1993; Iadecola et al., 1993; Regidor et al., 1993; Yan et al., 1996). Using infrared videomicroscopy, Cauli et al. (2004) also demonstrated that the GABAergic neurons not only contacted the neighboring penetrating microvessels, but that their dendrites and/or axonal branches reached for several blood vessels within an area that extended >100 μm away from the cell. In humans, processes of type 1 NADPHd-positive neurons are also intimately entwined with blood vessels in the cortex (DeFelipe, 1993; Garbossa et al., 2005) but their relative vascular and nonvascular distributions have not been investigated.

## Functional significance of the anatomical relationship between NOS interneurons and blood vessels

NOS interneurons are considered multifunctional as they release various neuromediators with different physiological effects. This multifunctionality requires a well-organized anatomy for a high spatial specificity in NVC. An example of this specificity is the finding of a distribution of an axonal plexus of densely NADPHd positive neurons around a restrictive group of microvessels in human temporal cortex (DeFelipe, 1993). NOS interneurons co-localize with the very powerful vasoconstrictor NPY in the cerebral cortex. Neurovascular associations have been reported for NPY in the human striate cortex (Berman and Fredrickson, 1992). Thus, the local release of NPY through the perivascular axonal plexus may produce a very localized vasoconstriction that will provide a spatial limitation to the vasodilation around the neuronal soma and dendrites (Estrada and DeFelipe, 1998). In contrast, NO possesses a volume of influence of up to 350 μm from a source point of production and may in effect dilate vessels within this radial length (Santos et al., 2012). Another possible role of NPY in the NOS interneuron-induced vascular response is to timely limit the vasodilation. Indeed, direct activation of a single nitrergic interneuron is sufficient to increase the diameter of a neighboring microvessel, but reversible dilatation is only induced by stimulation of a NOS neuron coexpressing NPY (Cauli et al., 2004).

The role of GABAergic transmission in the control of CBF also remains obscure, but anatomical and physiological evidences suggest that it could directly act on vessels to induce a vasodilation. GABA can induce intraparenchymal dilation through GABAa receptors located on vessels (Fergus and Lee, 1997). Glial cells also possess GABA receptors (Bureau et al., 1995). It is therefore possible that GABA modulates the vascular tone by acting on perivascular astrocytes. Consequently, the net effect of GABA on vascular tone stems from a very complex interaction with vessels, astrocytes as well as cortical interneurons and pyramidal neurons receiving GABAergic innervations.

Cortical NOS interneurons receive projections from the basalocortical Acetylcholine (Ach) and NO-synthesizing fibers as well as from the brainstem 5-hydrotryptamine (5-HT) afferents in both rat and human (Vaucher et al., 1997; Tong and Hamel, 1999). A large proportion (~ 30%) of NOS neurons receive both Ach and 5-HT innervations and 60% of these NOS containing neurons contact local blood vessels either with their proximal or distal neurites. Their neuronal processes extend long distances and contact a broad array of microvessels. NOS neurons thus appear exceptionally well positioned to relay Ach and 5-HT afferent information to blood vessels since they also exquisitely contact neighboring and remotely located blood vessels (Estrada and DeFelipe, 1998). Nevertheless, studies with 7-nitroindazole (7-NI), a selective nNOS inhibitor, failed to attenuate the CBF increase in response to basal forebrain activation (Zhang et al., 1995; Iadecola and Zhang, 1996). On the contrary, nonselective NOS inhibitors such as L-N^G^-nitroarginine (L-NNA) attenuate the CBF increase induced by stimulation of the basal forebrain (Raszkiewicz et al., 1992). Ach fibers directly contact cortical microvessels or the surrounding astrocytes in rat and man (Mesulam et al., 1992; Vaucher and Hamel, 1995; Tong and Hamel, 1999) and could as a result directly activate eNOS. The lack of an additive CBF effect of the coapplication of muscarinic receptor antagonist, atropine, and nonselective NOS inhibitor, L-NNA, on the cerebral cortex compared to L-NNA alone, suggest that Ach increases CBF possibly through eNOS activation (Zhang et al., 1995). In this context, the direct effect of Ach nerves on the vascular tone may encompass those mediated by nitrergic interneurons. However, in pathological situations where endothelial functions are altered, NO interneurons may significantly control CBF following basal forebrain stimulation.

## Neurally-derived nitric oxide and cerebrovascular regulation

### Resting cerebral blood flow

Many evidences suggest that NO plays a role in the cerebrovascular regulation. Various studies demonstrated that systemic or topical administration of non-selective NOS or selective nNOS inhibitors decrease resting CBF in many species. This effect is not observed with the iNOS inhibitor, aminoguanidine (Iadecola et al., 1995). Intraperitoneal injections of 7-NI, an *in vivo* inhibitor of the neuronal isoform of NOS, lower baseline CBF in unanesthetized rats (laser Doppler on the somatosensory cortex) (Montecot et al., 1997) (autoradiography in the barrel cortex) (Gotoh et al., 2001a) and anesthetized mice (laser Doppler on the somatosensory cortex) (Girouard et al., 2007, 2009), cerebral capillary flow in anesthetized rats (intravital microscopy on the right parietal cortex) (Hudetz et al., 1998), global CBF in anesthetized rats (autoradiography) (Cholet et al., 1997), and global CBF in cats (PET scan) (Hayashi et al., 2002a). The decreased cortical CBF ranges from about 10 to 60% depending on the 7-NI concentrations and the cortical areas and it is not accompanied by a decrease in glucose utilization (Kelly et al., 1995; Cholet et al., 1997; Hayashi et al., 2002b).

At resting state, arterioles exhibit slow spontaneous rhythmic diameter and blood flow changes of about 0.1 Hz defined as vasomotion. Vasomotion has been detected in cerebral arteries (Fujii et al., 1990; Filosa et al., 2004) and may be influenced by a variety of physiological factors including NO (Fujii et al., 1990). Mathematical models of myogenic mechanisms have demonstrated that vasomotion oscillations can be propagated locally and self-sustained within a group feeding of vessels (Behzadi and Liu, 2005). These models also suggest that a vessel with an oscillating diameter conducts more flow than a vessel with a static diameter (Meyer et al., 2002). Systemic administration of nonselective NOS inhibitors lead to the enhancement of oscillations within a common feeding vessel that are propagated to downstream branches (Griffith and Kilbourn, 1996; Behzadi and Liu, 2005). However, this phenomenon may be a consequence of the increase in systemic blood pressure and respiratory rate. The administration of a nNOS specific inhibitor or the local application of NOS inhibitors would give more specific information about the role of NO in the regulation of spontaneous vasomotion.

The CBF reduction in the presence of 7-NI is equivalent to the CBF attenuation induced by nonspecific NOS inhibitors which suggests that the neuronal isoform is responsible for the maintenance of resting cerebrovascular tone. Despite this, resting CBF evaluated with the hydrogen clearance method did not significantly differ between wildtype, nNOS- and eNOS-null mice (Atochin et al., 2003). This discrepancy may be explained by compensatory mechanisms in transgenic mice. Another argument for a neuronal specific effect on resting CBF is that the topical application of the specific NMDA receptor antagonist, MK801, reduces resting CBF to the same extent as 7-NI (Girouard et al., 2009). This is consistent with a nNOS role in the maintenance of resting CBF as no functional NMDA receptors in rat and human cerebromicrovascular endothelial cells has been identified (Morley et al., 1998). More importantly, isolated cerebral arteries in a number of species have not been found to dilate in response to application of glutamate or NMDA (Faraci et al., 1993; Simandle et al., 2005) and endothelial damage *in vivo* failed to affect NMDA-induced dilation (Domoki et al., 2002). Interestingly, NOS interneurons are spontaneously active. These spontaneous spikes are likely to be initiated by a baseline NMDA receptor activity detectable during subthreshold synaptic activation (Katona et al., 2011). It is thus possible that NOS interneuron spontaneous activity participates to maintain a certain level of resting CBF.

Anatomical and physiological evidences suggest the presence of nNOS in arterial smooth muscle. Neuronal NOS has been found in smooth muscle of cerebral vessels (Toda and Okamura, 2011) as well as in the common carotid artery (Brophy et al., 2000). In addition, 7-NI increases contractile response of carotid artery (Brophy et al., 2000). These results brought controversies about the origin of NO that contributes to the maintenance of resting CBF. However, in Brophy's study, 7-NI was applied on isolated arteries whereas 7-NI acts as a preferential nNOS inhibitor when administered systemically only (Babbedge et al., 1993). Using electron microscopy, Wang et al. (2005) did not observe any nNOS in vascular smooth muscle cells, endothelium, or glial processes.

Assuming that NO contributing to the resting CBF comes from neurons, the neuronal subtype responsible for its formation still has to be clearly identified. Although a clear participation of nitrergic nerves from the pterygopalatine ganglion has been demonstrated in the dilation of the middle cerebral artery and the posterior communicating arteries (Toda and Okamura, 2003), no studies have identified the specific neuronal NO origin that control parenchymal arteries tone. The use of Cre-dependent optogenetic transgenic mice for light induced activation and silencing different types of neurons will be necessary to clarify this issue (Madisen et al., 2012).

### Autoregulation

Cerebrovascular resistance decreases or increases in response to changes in transmural pressure and blood flow so that flow remains constant. Changes in resistance result from vasodilation and vasoconstriction of the pial and parenchymal vessels (Shapiro et al., 1971). Several studies have investigated the role of NO in the mechanisms involved in autoregulation. Nonspecific NOS inhibitors were administered while arterial pressure was decreased in a stepwise manner in order to test the role of NO in the lower limit of autoregulation. Although data coming from these studies were divergents the lower limit of autoregulation was raised in eNOS knockout mice (Huang et al., 1994) and remained normal in nNOS knockout mice (Huang et al., 1996). In addition, a study showing nonspecific NOS-induced autoregulation dysfunction failed to demonstrate similar effects with the nNOS inhibitor 7-NI (Toyoda et al., 1997) suggesting that eNOS is involved in the vasodilation in response to decreases in arterial pressure. To test the upper limit of autoregulation, arterial presure is gradualy elevated. Contrasting results emerged from studies using nonspecific NOS inhibitors. No effects have been observed with 7-NI (Hardy et al., 1999). Although many questions remain unresolved concerning the effect of NO in the control of autoregulation, there are no indications that (inter)neurons are involved in these mechanisms.

### Hypercapnia and hyperoxia

CBF is highly sensitive to alterations in arterial blood gases. For instance, increases in the partial pressure of arterial CO_2_ (PaCO_2_) and O_2_ (PaO_2_) provoke dilation and constriction, respectively. In several mammalian species including humans, it has been reported that blockade of NO synthesis with NOS inhibitors attenuates CBF responses to hypercapnia (Iadecola, 1992; Wang et al., 1992; Buchanan and Phillis, 1993; Pelligrino et al., 1993; Iadecola and Xu, 1994; Iadecola and Zhang, 1994; Sandor et al., 1994; Heinert et al., 1999) in the entire brain (Bonvento et al., 1994). The participation of NO is more important in mild hypercapnia (PaCO_2_ = 50–60 mm Hg) (Iadecola and Zhang, 1994). The cellular sources of NO during hypercapnia remain to be established but some evidences suggest a role for nNOS-derived NO in vascular responses to hypercapnia. Hypercapnia increases nNOS-derived NO in rat brain (Harada et al., 1997). Administration of 7-NI reduces the vasodilator and CBF responses to hypercapnia in anesthetized rats, suggesting that NO synthesized by nNOS participates in hypercapnic hyperemia (Wang et al., 1995; Okamoto et al., 1997). Endothelial denudation does not alter basilar and middle cerebral artery dilation in Japanese monkeys and Mongrel dogs in response to moderate hypercapnia (Toda et al., 1996). However, light/dye endothelial injury as well as NOS and soluble guanylate cyclase inhibition reduce hypercapnic cerebrovascular dilatation in anesthetized juvenile pigs, indicating that endothelial NO may play a significant role in the hypercapnic vasodilatation in this model (Willis and Leffler, 2001). Thus, mostly nNOS-derived NO seem to participate in the vascular response to hypercapnia while both eNOS and nNOS derived NO may participate in the autoregulation of the particular piglet cerebrovascular regulation.

The mechanisms by which NO modulates the hypercanic vascular response are not clear. However, hypercapnia provokes H^+^ accumulation and acidosis which, by converting Ca^2+^ waves to sparks, leads to the activation of BK(Ca^2+^) channels to induce dilation of cerebral parenchymal arteries (Dabertrand et al., 2012). This effect may be amplified by the modulating effect of NO on large conductance Ca^2+^-dependent K^+^ (BK) channels. This is consistant with the presence of hypercapnic vasodilation in isolated arteries in concert with NOS participation in the acidosis-induced vasodilation (Niwa et al., 1993) and the permissive role of NO in hypercapnic vasodilation (Iadecola and Zhang, 1994).

Hyperoxia causes a transient decrease in CBF, followed by a later rise. From mice lacking nNOS or eNOS and wild-type mice, Atochin et al. (2003) obtained evidences suggesting that cerebral vasodilatation induced by 60-min exposure to hyperbaric oxygen depends on both nNOS and eNOS. However, the underlying mechanisms remain to be investigated.

### Neurovascular coupling

Active neurons send various signals to astrocytes and blood vessels in order to increase CBF and obtain sufficient O_2_ and substrates. A number of studies have provided evidences that NOS inhibition attenuates the CBF increase in response to neuronal activity. Indeed, local application of NOS inhibitors suppresses the activity-dependent vasodilation by about 50% (Dirnagl et al., 1994; Irikura et al., 1994; Peng et al., 2004; Girouard et al., 2007; Kitaura et al., 2007). Systemic administration of 7-NI has also been shown to reduce the amplitude of NVC in different experimental paradigm of neuronal activation such as whisker stimulation and electric stimulation of mice forepaw (Cholet et al., 1996; Bonvento et al., 2000; Girouard et al., 2007; Liu et al., 2008). In both eNOS and nNOS-knockout mice, activity-dependent vascular changes were similar to those in control mice. However, in eNOS null mice, activity-dependent vasodilation was suppressed by NOS inhibitors (Ayata et al., 1996) while in nNOS knockout mice, NVC remained intact in the presence of NOS inhibitors (Ma et al., 1996). These data support the participation of neurogenic NO in NVC.

Reports on awake animals brought controversies about the role of NO on NVC. Studies with nonspecific NOS inhibitors, in which CBF was quantitatively determined in unanesthetized restrained animals, have failed to support such a role for NO (Sokoloff et al., 1992; Wang et al., 1993; Adachi et al., 1994). These results conclude that NVC is modulated by NO in anesthetized conditions only. However, in unanesthetized unrestrained rats, unspecific and specific nNOS inhibition decreases the vascular response to neuronal stimulation (Gotoh et al., 2001b). This discrepancy could be explained by the fact that immobilization of animals is a stressful condition that lowers paCO_2_, increases circulating catecholamines and therefore alter cerebrovascular regulation (Lacombe and Seylaz, 1984).

Another aspect that may mislead data interpretations is the potential effect of NO on neuronal activity (Lonart et al., 1992; Manzoni and Bockaert, 1993; Schuman and Madison, 1994; Jayakumar et al., 1999) that could explain its effect on NVC. Some studies have shown a decreased amplitude of somatosensory-evoked potentials induced by electrical stimulation of the forepaw or the sciatic nerve in the presence of NOS inhibitors (Ngai et al., 1995; Stefanovic et al., 2007). Other investigations demonstrated that NOS inhibition has no significant effect or a very slight effect on somatosensory evoked potentials (Lindauer et al., 1996; Burke and Buhrle, 2006; Hoffmeyer et al., 2007) or cerebral glucose activation (Cholet et al., 1997). Notwithstanding the observed effects on activated potentials, NOS inhibition was always accompanied by CBF reduction in *in situ* electrical response to neuronal supporting a role in the modulation of the vascular response.

Local NOS interneuron density varies among brain areas. Consequently, in each region, CBF may be differently regulated by NO. Cholet et al. reported that NO plays a role in NVC in the somatosensory area and the thalamus but less so in the trigeminal primary nucleus in rats under peri-oral somatosensory stimulation (Cholet et al., 1997). During vibrissal stimulation in unanesthetized rats, NOS inhibition with L-NAME attenuates the increase in CBF in the ventroposteromedial thalamic nucleus and 7-NI reduces the CBF in both the ventroposteromedial thalamic nucleus and the barrel cortex. NOS inhibitors did not significantly affect CBF in the spinal trigeminal nucleus and the principal sensory trigeminal nucleus (Gotoh et al., 2001a). Electrical somatosensory stimulation in the unilateral cat forepaw elicits an increase in CBF in the contralateral somatosensory cortex and the ipsilateral cerebellum which is attenuated by 7-NI (Hayashi et al., 2002b). These results are consistent with NOS interneuron distribution and strongly support the idea that NO regulating CBF comes from GABAergic neurons (Bertini et al., 1996; Mize et al., 1996).

CBF responses reflect the combined activities of cells and synapses that include both excitatory and inhibitory processes. Hence, depending on the afferent pathway and intensity, distinct population of neurons may be recruited. Electrical stimulation of two different pathways—cortico-cortical (transcallosal) or thalamocortical (infraorbital)—results in increased cortical CBF associated with the recruitment of distinct populations of interneurons and cyclo-oxygenase (COX)2 pyramidal cells (Enager et al., 2009). Analysis of double-immunostained cells with c-Fos and specific neuronal markers indicate that after 4 Hz thalamocortical stimulation, activation of COX2 expressing pyramidal cells and specific subtypes of inhibitory GABA interneurons that contain PV and SOM, a subset of the latter, are also known to contain NOS (Kubota et al., 1994). Transcallosal stimulation at 4 Hz sollicitates more COX2 pyramidal cells and less SOM interneurons (Enager et al., 2009). However, at 30 Hz, a larger fraction of SOM-NOS interneurons were activated together with a subset of VIP interneurons, but there was a silencing of those containing PV. Blood flow responses to these stimulations show a strong correlation between the CBF amplitude and the percentage of the SOM eNOS subset of GABAergic interneurons recruited (Enager et al., 2009).

The NO release from (inter)neurons is stimulated by glutamatergic neurotransmission (Faraci and Breese, 1993). Glutamate binds to its NMDA ionotropic receptors to produce NO by the activation of nNOS, an enzyme tethered to the NMDA receptor NR1 subunit receptor complex by the post-synaptic density protein-95 (Christopherson et al., 1999). This is consistent with the presence of nNOS in post-synaptic dendrites (Wang et al., 2005), NMDA R1 subunit mRNA or immunoreactivity in the majority of nNOS-immunoreactive neurons in rat cerebral cortex (Price et al., 1993). Ionotropic NMDA receptor stimulation results in Ca^2+^ influx, membrane depolarization, activation of nNOS, and the subsequent production of NO, which diffuses out of the neurons and acts on smooth muscle cells in local arterioles and pial arteries to cause vasodilatation (Faraci and Brian, 1995; Meng et al., 1995; Pelligrino et al., 1996; Garthwaite et al., 1989a; Girouard et al., 2009). NO may also directly act on pericytes surrounding terminal arterioles and capillaries (Hamilton et al., 2010).

The hypothesis that the nNOS-induced activation by NMDA receptors contributes to the increase in CBF is supported by the following observations: (1) NMDA increases NO production around arteries as demonstrated by the accumulation of NO degradation products in the cerebrospinal fluid surrounding the arteries (Domoki et al., 2002) or directly in the arteries (methemoglobin detection by spectroscopy) (Gonzalez-Mora et al., 2002) following NMDA application in the cortex and the simultaneous increase in CBF and NO production measured using microdialysis after NMDA perfusion in the striatum (Bhardwaj et al., 2000). (2) Both dilation and NO increases induced by NMDA are reduced by treatment with nonspecific and specific nNOS inhibitors in various animal species (Faraci and Breese, 1993; Faraci and Brian, 1995; Meng et al., 1995; Bhardwaj et al., 2000; Gonzalez-Mora et al., 2002). (3) These effects are abolished by the administration of the sodium channel blocker tetrodotoxin (TTX), an agent which does not usually affect the endothelium (Faraci and Breese, 1993; Pelligrino et al., 1996) whereas endothelial stunning by phorbol 12,13-dibutyrate leaves NMDA-induced dilation intact (Domoki et al., 2002). Exception has been found in the piglet which seems to be the unique model expressing ionotropic glutamate receptors in the endothelium and where dilator responses of cerebral resistance vessels to glutamate were reported to be intact despite the presence of TTX (Leffler et al., 2006). (4) Tissue plasminogen activator (tPA) is critical to the full expression of the flow increase evoked by activation of the mouse whisker barrel cortex and increases NMDA-induced NO production by increasing nNOS phosphorylation state (Park et al., 2008).

Differential recruitment of ionotropic glutamatergic receptors (GluRs) seems to result from different stimulation frequencies. Indeed, ionotropic nNOS inhibition reduces CBF responses to all stimulation frequencies by 50% (Dirnagl et al., 1993; Hoffmeyer et al., 2007) while NMDA receptor blockade attenuates CBF responses only at high frequencies. This suggests that nNOS activity may be stimulated by NMDA-independent mechanisms. This could involve gating of Ca^2+^ permeables AMPA or kainate receptors as well as opening voltage sensitive Ca^2+^ channels (Goldberg and Yuste, 2005). Application of kainate and AMPA receptor agonists also results in NO production (Garthwaite et al., 1989b; Bhardwaj et al., 1997a) and dilation of cerebral arteries in all species studied when applied to the cerebral cortex. Dilation to kainate is attenuated by inhibition of NOS (Faraci et al., 1994). It also appears that antagonists or blockers of heme oxygenase and COX reduce dilation to kainate (Bari et al., 1997; Ohata et al., 2006) or to the kainate receptor agonist, (*RS*)-2-amino-3-(3-hydroxy-5-*t*-butylisoxazol-4-yl) propionic acid (ATPA) (Robinson et al., 2002). Arterial dilation to AMPA is not restricted by superfusion of NOS inhibitors but is attenuated by adenosine A(2A) and A(2B) receptor antagonists and the HO inhibitor chromium mesoporphyrin (Ohata et al., 2006).

Although glutamate has the potential to induce vasodilation though activation of any of the three ionotropic glutamate receptors, this effect mostly involves NMDA receptors. Thus, MK-801, a selective NMDA receptor antagonist, blocks almost all of the cerebral dilator response to glutamate or NMDA in rabbits, piglets, and rats (Faraci and Breese, 1993; Meng et al., 1995; Pelligrino et al., 1995, 1996; Girouard et al., 2009). This preference for the NMDA receptors in the cortex may be due to their location in the cerebral cortex or due to their greater affinity for glutamate than kainate or AMPA receptors.

In the mouse somatosensory cortex, serotonin 5-HT_3A_ receptors have been identified in neurogliaform like regular spiking neurons corresponding to GABAergic neurons expressing NPY and NOS (Vucurovic et al., 2010; Markwardt et al., 2011). This receptor, which is the only ionotropic serotonergic receptor (Chameau and van Hooft, 2006), mediates fast serotonin-induced excitation (Ferezou et al., 2002). Perrenoud et al. (2012a) found that activation of 5-HT3A-expressing interneurons mostly induces NO-mediated vasodilation and, less frequently, NPY-mediated vasoconstriction. The same study also demonstrated that these effects are absents in Pet-1 knock-out mice which display a drastic depletion of cortical serotoninergic fibers (Kiyasova et al., 2011) supporting NPY/NOS interneuron-dependent vasomotor effects rather than presynaptic 5-HT3 activation of serotonergic axons originating from the raphe nuclei. According to Perrenoud et al. (2012a) activation of the serotonin 5-HT3A receptors triggers NPY and NO release by a mechanism independent of action potential generation, but rather by direct Ca^2+^ influx which may induce Ca^2+^ release from intracellular stores.

NO is one of the vasodilator that diffuses the most rapidly (Wood and Garthwaite, 1994) and its release, in the context of NVC, mostly depends on nNOS activation. The release of NO from neurons depends on intracellular Ca^2+^ elevation similar to other vasoactive mediators derived from neurons (Lauritzen, 2005) and astrocytes (Straub and Nelson, 2007). Thus, a fast activation of nNOS would occur following Ca^2+^ influx after fast neuronal depolarization (10–12 ms) (Petersen et al., 2003) and/or activation of ionotropic receptors while a slower release may be induced through the activation of metabotropic receptors leading to Ca^2+^ release from intracellular stores (Perea and Araque, 2005). Thus, hypothetically, NO may account for the initiation and the maintenance (up to 15 min) of the vasodilatatory response in NVC. This premise is supported by the following observations: (1) NO concentrations increase at the beginning of the stimulation and remain elevated during at least a 2 min duration of neuronal stimulation (de Labra et al., 2009); (2) CBF responses to 1 s stimulation of the mouse hindpaw is reduced by about 50% in nNOS knockout mice or after topical application of L-NA (Kitaura et al., 2007); (3) Topical application of L-NNA dampers the entire NVC; (4) Systemic administration of 7-NI attenuates NVC during long stimulus of more than 60 s (Dirnagl et al., 1993, 1994; Ngai et al., 1995; Lindauer et al., 1999; Peng et al., 2004). NO can either act directly on smooth muscle cells through the guanylate cyclase/cGMP system or indirectly by having a permissive role. In the cerebral cortex, the reduction of the magnitude of the NVC reduced by NOS inhibition could be restored by the normalization of basal NO levels with the infusion of the NO precursor, L-arginine NO (Northington et al., 1992; Lindauer et al., 1999). This effect is not observed after restoration of basal cGMP levels. These observations suggest that, in the cortex, NO is a modulator rather than a mediator of NVC (Iadecola et al., 1994; Lindauer et al., 1999). However, the vasodilation induced by NMDA is completely dependent of nNOS, guanylate cyclase, and protein kinase G (Girouard et al., 2009) which indicates that NO has an obligatory role in the NMDA-induced dilation and thus possibly in the fast ionotropic dependent CBF response to neuronal activation.

In summary, it seems that an initial rise of intracellular NO concentration in interneurons following activation of the glutamatergic or serotoninergic ionotropic receptors is responsible for the initiation of the vascular response whereas during long stimulation, NO would have a permissive role for other mediators to increase CBF.

### Role of astrocytes in the no modulation of neurovascular coupling (Figure 1)

Astrocytes are key elements in the neural activity-dependent regulation of vascular tone. Anatomically, astrocytes surround most of the arteriolar and capillary abluminal surface with their endfeet and are uniquely positioned between synapses and vessels. This specific arrangement led to the concept of a neuron-astrocyte-vasculature tripartite (Vaucher and Hamel, 1995). Functionally, astrocytes can integrate neurotransmitters signals from thousands of synapses (Bushong et al., 2002) and relay this information to the arterioles (Metea and Newman, 2006). Surely, results obtained in cortical brain slices show a temporal link between increases in Ca^2+^ in the astrocyte and the subsequent vasodilation of neighboring arterioles (Zonta et al., 2003a). Then, using two-photon microscopy, Nedergaard's team (Wang et al., 2006) confirmed *in vivo* the physiological involvement of astrocytic Ca^2+^ increase in NVC. In accordance with the functional studies, metabotropic GluRs blockers attenuate the astrocytic Ca^2+^ signal indicating that astrocytes sense and get activated by glutamate. The involvement of astrocytes is likely to occur in the late phase of NVC. Indeed, Calcinaghi et al. (2011) reported that pharmacological blockage of mGluR5 and mGluR1 mostly expressed on astrocytes (Porter and McCarthy, 1996) does not affect NVC in the somatosensory cortex of adult rats on brief whisker stimulation of 4 and 24 s. Whisker stimulation *in vivo* induces CBF increase after 600 ms (Devor et al., 2003) while it activates astrocytes with a latency of 3 s after the stimulus (Wang et al., 2006). *In situ* electrical stimulation within the barrel triggered astrocyte Ca^2+^ transients, which peaked within 1–2 s after stimulation (Schipke et al., 2008). These data strongly suggest that although astrocytic Ca^2+^ rises early after neuronal activation, astrocytic metabotropic activation does not play a significant role in the onset of CBF but is essential for the maintenance of the hemodynamic response during NVC. In addition, the lack of an astrocytic Ca^2+^ rise when neuronal activity is blocked by TTX, indicates that neurons and astrocytes act in series during the delayed phase of NVC.

**Figure 1:**
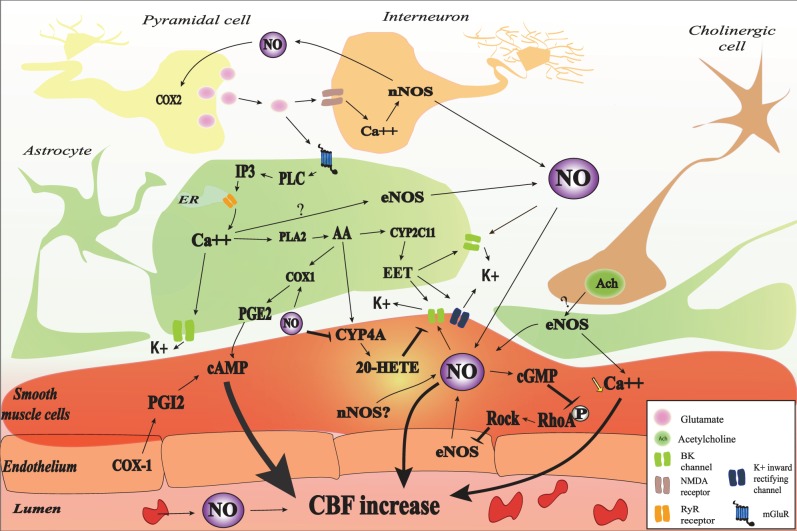
**Nitric oxide (NO) interactions in neurovascular coupling (NVC).** NO may be released from red blood cells, endothelial cells, smooth muscle cells, astrocyte, interneurons, and some pyramidal cells. NO may be involved in the initiation of NVC through a cyclic guanosine monophosphate (cGMP)-dependent pathway and may modulate NVC probably by interaction with different cGMP-independent pathways. In astrocyte, glutamate released from pyramidal cell binds to the metabotropic glutamate receptor (mGluR) which lead to IP_3_ formation and the subsequent release of Ca^2+^. The increase in intracellular Ca^2+^ activates BK channel and catalyses the phosphorylation of arachidonic acid (AA) by phospholipase A_2_ (PLA_2_). AA metabolites (PGE2, 20-HETE, and EET) will mediate cerebral blood flow (CBF) through different pathways. NO may interact with different astrocytic pathways such as Ca^2+^ release, Ca^2+^-activated K^+^ channel (BK) opening, and COX1 activity. NO can also decrease 20-HETE formation through the inhibition of CYP4A. Vascular smooth muscle cells are the final integrators of NO-induced signals coming from the endothelium, the blood, neurons, and astrocytes. In the SMC, NO directly mediate vasodilation through the increase in cGMP-PKG activity and the increase of BK channel opening probability and the subsequent hyperpolarization as well as the inhibition of RhoA phosphorylation. The contractile state of VSMC is mainly determined by the phosphorylation level of myosin light chains (MLC). MLC is phosphorylated by the Ca^2+^/calmodulin-dependent MLCK and dephosphorylated by the Ca^2+^-independent MLCP. NOS may be inhibited, in turn, by RhoA kinase (ROCK).

Some evidences support the hypothesis that NO derived from nNOS participates in the mGluRs induced CBF increase. The mGluR1 and five agonist, *trans*-1-amino-1,3-cyclopentanedicarboxylic acid (ACPD), is capable of increasing NOS activity *in vivo* through an IP3-dependent mechanism (Bhardwaj et al., 1997b). In addition, nNOS inhibition strongly attenuates the CBF increase to the mGluR1 activation with the agonist (S)-3,5-dihydroxyphenylglycine (DHPG) during the first 10–15 min of superfusion while it does not exert any significant effect after 15 min (Liu et al., 2011). These data suggest that NO interact with different astrocytic pathways instead of acting directly on smooth muscle cells. In the next paragraphs, we will describe the possible interaction of NO with different pathways involved in NVC.

### Reported effects of no on astrocytic Ca^2+^ signaling, BK and PLA_2_ pathways

For decades, investigators assumed that NO originating from neurons diffuses to smooth muscle cells to induce a vasorelaxation. However, astrocytes are anatomically closer to neurons and nNOS than smooth muscle cells. In addition, an increased cGMP production has been observed in astrocytes in response to exogenous NO as well as to glutamate and NMDA induced NO production (Malcolm et al., 1996). Willmott et al. (2000a) demonstrated that NO-PKG signaling is coupled to Ca^2+^ mobilization in isolated glial cells. In many cellular types, it has been demonstrated that NO induces Ca^2+^ mobilization (Publicover et al., 1993; Willmott et al., 1995a,b; Clementi et al., 1996) through the activation of the cGMP-PKG and ADP-ribosylcyclase and subsequent increase in synthesis of the potent Ca^2+^ ryanodine receptor (RYR) dependent mobilizing agent cyclic ADP-ribose (Galione et al., 1991; Willmott et al., 1995b; Clementi et al., 1996) or the direct nitrosylation of regulatory thiol groups of RYR (Stoyanovsky et al., 1997).

Cytoplasmic and endoplasmic reticulum (ER) Ca^2+^ are important determinant of IP3-mediated Ca^2+^ release, which delivers local Ca^2+^ in astrocytic endfeet for BK channel activation in the endfeet. The release of K^+^ into the arteriolar space acts on smooth muscle inward rectifying K^+^ channels to hyperpolarize the smooth muscle cell membrane, lower arteriolar smooth muscle [Ca^2+^] and thereby cause vasodilation (Straub et al., 2006). In several tissues, the cGMP/PKG pathway activates BK channels (Archer et al., 1994; Alioua et al., 1995; Carrier et al., 1997). BK channel activity is regulated by a variety of signaling molecules, including intracellular Ca^2+^ ([Ca^2+^]_i_) (Kume et al., 1989), protein kinases (Robertson et al., 1993), tyrosine kinases (Alioua et al., 2002), cytochrome P-450 metabolites of arachidonic acid (Zou et al., 1996), and heme (Tang et al., 2003). BK channels are also directly activated by O_2_, CO, and NO as demonstrated in cell-free membrane patches isolated from the intracellular medium (Bolotina et al., 1994). Thus NO may modulate BK channel opening directly or by inducing cytosolic Ca^2+^ increase. BK channels are typically composed of pore-forming α subunits that are encoded by the *Slo1* (or *KCNMA1*) gene, and accessory ß subunits that modulate channel gating (Tanaka et al., 2004). Stimulatory effect of NO on BK channels is likely mediated by the β subunits of BK channel complex. NO elevates BK channel Ca^2+^ sensitivity (Tanaka et al., 2004), enhancing the effective coupling of Ca^2+^ to BK channels (Jaggar et al., 2002).

**Figure 2:**
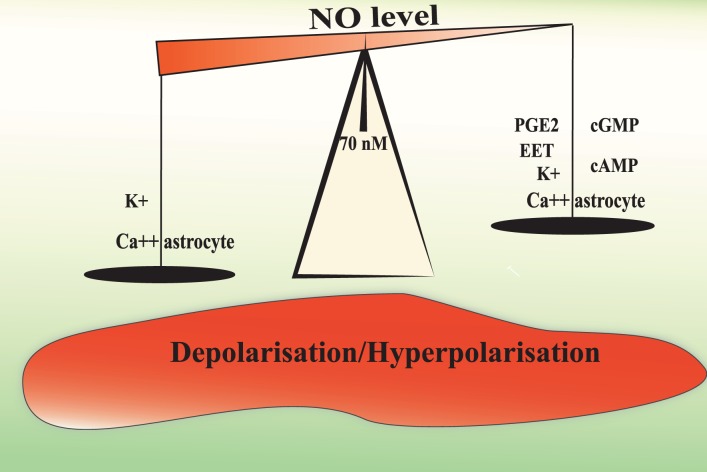
**Hypothetical mechanisms underlying the biphasic effect of NO in the modulation of NVC.** A moderate or physiological NO increases the release of vasorelaxing factors from the astrocyte and depolarise smooth muscle cells to increase cerebral blood flow. NO levels above 70–80 nM may increase astrocytic Ca^2+^ mobilization and BK channel opening probability to a level that will increase the perivascular K^+^ ion concentrations over 20 mM. Such a high K^+^ concentration induces smooth muscle membrane depolarization and the consequent vasoconstriction.

The level of Ca^2+^ in astrocytic endfeet determines the nature of the vascular response with moderate elevations causing dilation and larger elevations causing constriction. The constrictive responses in preconstricted arteries in brain slices incubated in artificial cerebrospinal fluid with stable levels of oxygen seem to be entirely dependent on BK channels (Girouard et al., 2010). In acute mammalian retina, high doses of the NO donor SNAP (100 μM) blocks light-evoked vasodilations or transforms vasodilations into vasoconstrictions. This effect is prevented with PTIO (a NO scavenger, 2-pyenyl-4,4,5,5-tetramethylimidazoline-3-oxide-1-oxyl). In the presence of L-NAME, light stimulation evokes only vasodilations. All arterioles dilate in response to light when NO is lower than 70 nM. At NO concentrations between 80 nM and 1 μM, light-evoked responses switch from dilation to constriction (Metea and Newman, 2006). Electrical stimulations in brain slices raises NO concentration up to 100 nM as measured by electrodes (Shibuki and Okada, 1991) and levels up to 1–4 μM have been measured in rat brain *in vivo* during ischemia and reperfusion (Malinski et al., 1993). In this context, the NO induced vasoconstriction is probably pathological. The NO capacity to mobilize astrocytic Ca^2+^ and to increase BK channels opening probability may explain why exogenous NO could transform vasodilation into vasoconstriction and support the idea of a modulating role of NO in NVC (Figure 2).

Astrocytic cytosolic Ca^2+^ induces arachidonic acid release (Alkayed et al., 1997), which serves as a substrate for the synthesis of epoxyeicosatrienoic acids (EETs) by CYP2C and CYP2J epoxygenases in astrocytes (Alkayed et al., 1996, 1997; Nithipatikom et al., 2001; Peng et al., 2004), prostaglandin E2 (PGE2) by COX1 in astrocytes (Zonta et al., 2003b), and the synthesis of 20-HETE by CYP4A enzymes in cerebral vascular smooth muscle (Dunn et al., 2008). Astrocyte-derived EETs promote increases in astrocyte Ca^2+^ and opening of astrocytic BK channels (Yamaura et al., 2006). They may also diffuse from astrocytes to the adjacent smooth muscle cells to open BK channels and hyperpolarize vascular smooth muscle cells (Alkayed et al., 1997). Specific inhibitors of EET synthesis, MS-PPOH and miconazole, or antagonist of EET receptors, 14,15-EEZE, show that EETs contribute to 40–60% of the CBF response to sensory stimulation (Liu et al., 2011). In contrast, 20-HETE depolarizes smooth muscle cells by inhibiting the opening of K^+^ channels (Lange et al., 1997) and enhances Ca^2+^ influx through voltage-dependent Ca^2+^ channels (Gebremedhin et al., 1992). The 20-HETE inhibitor HET0016 blocks the vasoconstriction elicited by electrical activation in rat brain slices (Hama-Tomioka et al., 2009). *In vivo*, HET0016 attenuates the CBF response during the first 5 min of activation of the group I mGluRs with DHPG superfusion, suggesting that 20-HETE might serve as a vasodilator during the first phase of the group I mGluR-induced vasodilation (Liu et al., 2011). This effect may be explained by the conversion of 20-HETE to the vasodilator 20-hydroxy-PGE by COX (McGiff and Quilley, 1999). When mGluR activation is prolonged beyond 30 min, the inhibition of 20-HETE synthesis prevents the decrement of the CBF suggesting that this mediator may limit CBF increase periods (Liu et al., 2011). The other arachidonic acid metabolite, PGE2 released from astrocytes may travel to smooth muscle cells and induce vasodilation by binding to EP_4_ prostaglandin receptors (Davis et al., 2004), which increase the activation of protein kinase A by cyclic AMP and as a result decrease the phosphorylation of the myosin light chain (Takata et al., 2009).

NO can affect vasomotor responses by inhibiting ω-hydroxylase, the synthetic enzyme for the vasoconstrictor 20-HETE (Roman, 2002). It also enhances prostaglandin production by COX1 (Fujimoto et al., 2004). The concomitant inhibition of the synthesis of the vasoconstrictor 20-HETE and COX1 expressed in astrocytic endfeet (Gordon et al., 2008) stimulation by NO may underlie a significant fraction of the dilating effect of NO (Sun et al., 2000) and may be another mechanism by which NO modulates NVC in a cyclic GMP-independent manner. Also, by counteracting the decrement of CBF by 20-HETE at the end of the stimulation, NO could increase the length of the CBF response.

NO can also enhance the catalytic activity of COX2 in the brain (Salvemini, 1997). However, although COX1 is present in astrocytes, COX2 is rather present in neurons post and presynapticaly to nNOS neurons (Bidmon et al., 2001). Anatomical evidences suggest a close interaction between COX2 and NO. Firstly, COX2 and nNOS are both prevalent in layer I and III, IV (Degi et al., 1998). Secondly, COX2 are detected in dendrites that are lightly immunolabeled for nNOS. Both COX2 and nNOS are close to walls of penetrating arterioles and capillaries and separated by thin glial processes (Wang et al., 2005), which indicate that their interaction may play a role in the regulation of NVC. However, physiological studies have shown an additive effect with unspecific COX and NOS inhibitions (Kitaura et al., 2007), suggesting independent pathways.

### Retrograde vasodilatation (Figure 3)

During NVC, local vasodilation must be associated with dilation of upstream pial arteries (Duling et al., 1987). This has been observed in several brain areas. For example, activation of whisker barrel cortex increases vascular diameter in pial arterioles that are several hundred micrometers away from the site of activation (Ngai et al., 1988; Cox et al., 1993; Erinjeri and Woolsey, 2002). Several possible mechanisms have been proposed for this complex and coordinated chain of events, including widespread neurovascular innervation, propagation of astrocytic Ca^2+^ waves and “intramural” signaling within the vascular wall. Vascular smooth muscle cells probably receive and integrate signals coming from neurons, glia, endothelial cells as well as mechanical forces. Indeed, neurons release NO, glutamate, and ATP that have been shown to play a role in the astrocytic Ca^2+^ wave propagation. NO is a good candidate for the propagation of neuronal signals since it can diffuse very rapidly and propagate to a distance of up to 100 μM. NO can also potentiate the propagation by amplifying the astrocytic signals such as Ca^2+^ waves and ATP and glutamate release from astrocytes. Indeed, the addition of puffs of aqueous NO induces large astrocytic Ca^2+^ transients that propagate to a group of up to 20 cells (Willmott et al., 2000b). This effect seems to be mediated through PKG and RyR-linked Ca^2+^ release as well as gap junction (Bolanos and Medina, 1996; O'Donnell and Grace, 1997). Astrocytes are known to have a vesicular pool of glutamate (and possibly ATP) that is rapidly exocytosed in response to agonists such as NO that raise intracellular Ca^2+^ (Maienschein et al., 1999; Pasti et al., 2001). Nanomolars levels of ATP can act via extracellular mediators and may carry information between astrocytes (Scemes, 2000). NO is also known to cause rapid glutamate release from neurons (Meffert et al., 1994). Thus NO may propagate the neuro-astrocytic signals directly by increasing intracellular concentrations of Ca^2+^ or through the release of ATP and glutamate. ATP may also be metabolized and produce adenosine, a potent vasodilator. Adenosine has long been implicated as a mediator of NVC (Ko et al., 1990; Dirnagl et al., 1994). Indeed, adenosine A_2A_ receptors have a role in dilation of upstream pial arterioles during neuronal activation or direct activation of GluRs (Iliff et al., 2003; Ohata et al., 2006). In addition to acting on vascular adenosine receptors, adenosine could act on astrocytes, which also express various types of adenosine receptors (Fields and Burnstock, 2006). Activation of A_2B_ receptors can increase intracellular Ca^2+^ in astrocytes and might participate in the propagation of increased Ca^2+^ throughout the astrocyte processes (Pilitsis and Kimelberg, 1998). Ordinarily, an increase in Ca^2+^ is associated with release of ATP through connexin hemichannels, and A_2B_ receptors potentiate the ATP-evoked Ca^2+^ response (Jimenez et al., 1999; Alloisio et al., 2004). Finally, the release of GABA from interneurons restricts the astrocytic response to the barrel column (Benedetti et al., 2011).

**Figure 3:**
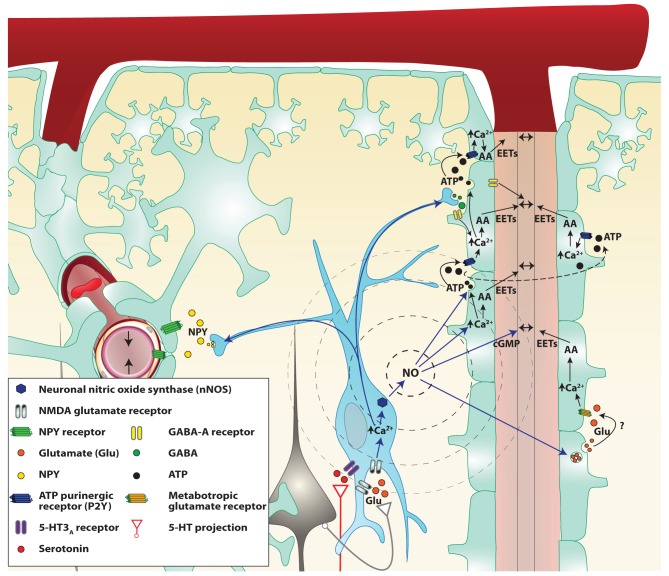
**Proposed mechanisms by which NO interneurons may propagate the signal in the cortex.** Two parenchymal descending arteries (left and right of the figure) are branching from a pial artery. Astrocytes (light green cells) cover descending arteries with their specialized projections: the astrocytic endfeet, with the exception of the pial artery and parenchymal arteries portion separated by the Virchow–Robin space. Following local neuronal activity, pyramidal cells (black cells) stimulate nitrergic interneurons (blue cell), through their glutamate NMDA receptors. The influx of calcium (Ca^2+^) in the cell allows activation of the neuronal NO synthase (nNOS) and the production of NO. NO diffuses freely through cell membranes and reaches the astrocytic endfeet where it could modulate the release of various vasoactive metabolites. NO may be involved in retrograde vasodilation either through astrocyte signaling modulation or by acting directly on smooth muscle cells. Indeed NO can increase the release of ATP and glutamate from astrocytes. ATP released from vesicles goes from one astrocyte to the next stimulating P2Y purinergic receptors, creating a Ca^2+^ wave. These Ca^2+^ waves enhance the production of arachidonic acid (AA) derived vasodilation mediators like epoxyeicosatrienoic acids (EETs) or activate large conductance Ca^2+^-dependent K^+^ channels thus causing arterial vasodilation. NO also directly increases astrocytic Ca^2+^ and might therefore facilitate the production of vasodilators. Finally, NO binds to smooth muscle guanylate cyclase and increases the cGMP levels. Stimulated nitrergic interneurons also release GABA which could act either on astrocytes or smooth muscles GABA-A receptors to cause vasodilation. However, GABA seems to restrain the astrocytic signal propagation. Simultaneously, Neuropeptide Y (NPY) is released most probably to a remote vascular bed (on the left of the illustration) to cause a vasoconstriction and concentrate the blood flow close to the neuronal activation.

Endothelial and smooth muscle cells in the brain are connected by homocellular gap junctions and can propagate vasodilation in a retrograde fashion (Dietrich et al., 1996; Sokoya et al., 2006). Local vasodilation increases flow velocity in upstream branches which, due to increased shear stress, leads to the local release of endothelium-dependent vasodilators (Busse and Fleming, 2003). These vasodilators relax the larger arteries and amplify the increase of flow, but there is limited evidence supporting this possibility in the neocortical microcirculation. The hyperpolarization of vascular smooth muscle travels rapidly through gap junctions for millimeters (de Wit, 2010) or within the endothelial tissue through the small (KCa 2.3; KCNN3)-and intermediate (KCa 3.1; KCNN4)-conductance Ca^2^^+^-activated K+ channels (SKCa/IKCa) (Behringer and Segal, 2012).

## What is the specific role of interneuron-derived no in the control of CBF among other sources?

There are multiple sources of NO and all cell types of the brain can produce NO. NO derives from three NOS isoforms as well as from nitrite. In the central nervous system, the nNOS isoform is present in interneurons, some pyramidal cells as well as nitrergic nerves originating from pterygopalatine ganglia although their extraneuronal presence is still a matter of controversies. There are at least three nNOS alternative-splice variants: nNOSα, nNOSβ, and nNOSγ (Putzke et al., 2000; Saur et al., 2000). The mitNOS corresponds to the nNOSα variant and could be inhibited by 7-NI. In addition, only the nNOSα variant is knocked out in the nNOS null mice (Putzke et al., 2000), which means that these mice lack both neuronal and mitochondrial NOS. Neuronal NOSα null mice are resistant to NMDA-induced neurotoxicity (Dawson et al., 1996; Ayata et al., 1997). In a culture of rat hippocampal neurons, it has been demonstrated that nNOS is totally localized into mitochondria and activated by NMDA (Marks et al., 2005). Therefore, it is very difficult to conclude the source of NO using nNOS transgenic mice or 7-NI. Since NOS interneurons are very specifically localized and serve as a relay to pyramidal neurons, we could hypothesize that their primary function is the control of NVC and local resting CBF rather than global CBF changes such as in autoregulation or responses to (Ulker et al., 2009) systemic changes in paCO_2_ or paO_2_. However, the subcellular localization of NOS in interneurons remains controversial (Mizukawa et al., 1988; Wolf et al., 1992; Aoki et al., 1993; Wiencken and Casagrande, 2000).

Although, we already mentioned studies about the role of NOS isoforms in resting CBF, autoregulation as well as responses to paCO_2_ or paO_2_ variations, it is interesting to stress out the possible involvement of NO from blood cells in these mechanisms. Although blood cells can produce a considerable amount of NO, this aspect is rarely considered in neurovascular studies. Shear stress, defined as the tangential forces acting on the luminal surface of the vessel as a result of flow, could activate eNOS in the endothelium as well as red blood cells NOS which is very similar to eNOS (Ulker et al., 2009). In this regard, both endothelial and red blood cells would play a role in CBF autoregulation. Interestingly, red blood cells also contain functional NMDA receptors, which upon activation increase NOS-dependent NO production (Makhro et al., 2010). This may explain why resting CBF is decreased in the presence of the NMDA inhibitor MK-801 although the effect of 7-NI on red blood cell NOS remain to be assessed.

Another important source of NO in the brain is nitrite. However, nitrite was recently found to have a direct effect on resting CBF in a rat model (Rifkind et al., 2007) and on the CBF in response to hypercapnia and hypoxia in human (Peebles et al., 2008). Nitrite concentrations in different mammalian tissues are in general in micromolar range of 0.5–20 μM (Samouilov et al., 2007; Feelisch et al., 2008). Nitrite can be converted into NO through the nitrite reductase activity of deoxyhemoglobin (Cosby et al., 2003), xanthine oxidase (Li et al., 2008), aldehyde oxidase (Li et al., 2009), carbonic anhydrase (Aamand et al., 2009), and all isoforms of NOS (Mikula et al., 2009).

Overall, anatomical and physiological studies support a role of NOS interneurons in NVC while other NOS containing cells or nitrite seem to be rather responsible for the control of autoregulation or the responses to hypercapnia and hypoxia. Nevertheless, the development of specific NOS inhibitors is necessary to bring appropriate answers to questions related to the origin of NO in CBF regulation.

## No and functional braing imaging

Functional magnetic resonance imaging (fMRI) is often used as a marker of changes in neuronal spiking activity. In reality, fMRI detects CBF through the arterial spin label method or blood oxygenation levels (BOLD) which reflects the shift of the ratio between the local concentration of oxygenated and deoxygenated hemoglobin due to increased oxygen extraction and increased oxygen supply. Unfortunately, the relationship between hemodynamic changes and the actual underlying response is still poorly understood. Anatomical and physiological data about NOS interneurons strongly suggest a non-linear relationship between neuronal activity and fMRI signals. In fact, NOS interneurons account for 0.3% of all cortical neurons but for about 50% of the vascular response to neuronal stimulation. In addition, the inhibition of nNOS with 7-NI clearly reduces the BOLD response upon electrical forepaw stimulation while the somatosensory-evoked potentials are still clearly detectable and only slightly reduced in amplitude (Burke and Buhrle, 2006). Overall, these studies strongly support the concept of a non-linear relationship between the BOLD-fMRI signal and neuronal stimulation (Membre et al., 1997; Yang et al., 2000).

However, interneurons may act as “local integrators” of cortical activity and therefore have important implications for the resulting neuronal activity and functional brain imaging (Buzsaki et al., 2007). The resulting neuronal activity correlates with increases in local field potentials rather than spikes. For example, if the balance between excitation and inhibition does not lead to the firing of action potentials, as demonstrated in the visual cortex, the CBF response correlates with local field potentials but not with spikes. Thus, local field potentials reflect synchronized or correlated neuronal activity and are associated with incoming input and local processing (Logothetis et al., 2001). So, the CBF increase best reflects signal processing by interneurons and electrophysiological events that do not result in spikes. Nonetheless, the majority of studies suggest that the relationship between CBF or BOLD signal and local field potentials is non-linear. Consequently, fMRI signals should be carefully interpreted as a non-linear result of local field potential and the significance of NOS interneurons in this signal remain to be elucidated.

## Conclusion

NOS interneurons are strategically positioned close to blood vessels and seem to be involved in the control of CBF. In NVC, the mechanisms by which NO intervenes are particularly complex. Actual data suggest that NO from interneurons play a cGMP-dependent mediator role in the initiation of the vascular response to neuronal activation followed by a cGMP-independent modulatory role of astrocytic signals, which could lead to a vasodilation at moderate concentrations or a vasoconstriction at higher concentrations. NO from different sources also seems to coordinate the signal propagation of vasodilation upstream as well as the length of the response. The involvement of inhibitory NOS interneurons in NVC implies that the relationship between neuronal activity and CBF is non-linear. Therefore, a better understanding of the role of NOS interneurons in the control of CBF will allow a better modelization and interpretation of BOLD signal used in fMRI studies.

## Future directions

Since the discovery that NO is a powerful vasodilator, we have made many observations to support its pivotal role in the cerebrovascular regulation. However, a complete understanding of the origin of NO and the pathways by which it controls the vascular tone is still missing. Additional studies will have to be conducted to further determine how NO intervene in NVC, how it is released upon activation and how it interact with astrocytes. As pharmacological blockers show evident limitations when it comes to cell-type specificity, new techniques to unveil the direct involvement of NOS expressing interneurons in NVC have yet to be developed. A few transgenic mice models like the Arx (Colombo et al., 2007; Price et al., 2009) and the Tsc1 (Fu et al., 2011) mutant mice, present a diminished expression of NPY interneurons in the cortex. It would be interesting to verify whether CBF regulation is impaired in these models and what proportion of the remaining interneurons expresses nNOS. As these transgenic mice model might present compensatory mechanism and reorganization of the neural circuitry, more targeted approaches might be needed. Recently, a new method for specific elimination of nNOS expressing interneurons in acute slices using induced photo-toxicity has been elaborated (Shlosberg et al., 2012). However, the most promising tool will be conditional transgenic optogenetic silencing using Cre driver lines allowing the investigations of neural circuit function with unprecedented reliability and accuracy.

### Conflict of interest statement

The authors declare that the research was conducted in the absence of any commercial or financial relationships that could be construed as a potential conflict of interest.
